# Performing Sparse Regularization and Dimension Reduction Simultaneously in Multimodal Data Fusion

**DOI:** 10.3389/fnins.2019.00642

**Published:** 2019-07-03

**Authors:** Zhengshi Yang, Xiaowei Zhuang, Christopher Bird, Karthik Sreenivasan, Virendra Mishra, Sarah Banks, Dietmar Cordes, the Alzheimer's Disease Neuroimaging Initiative, Michael W. Weiner

**Affiliations:** Author Affiliations: UC San Francisco; University of Southern California; UC San Francisco University of Southern California Mayo Clinic, Rochester Mayo Clinic, Rochester; UC Berkeley; U Pennsylvania; USC; UC Davis; Brigham and Women's Hospital/Harvard Medical School Indiana University Washington University St. Louis University of Pennsylvania; Prevent Alzheimer's Disease 2020 (Chair) Siemens; Alzheimer's Association University of Pittsburgh Washington University St. Louis Cornell University; Albert Einstein College of Medicine of Yeshiva University; AD Drug Discovery Foundation; Acumen Pharmaceuticals; Washington University St. Louis; Northwestern University; National Institute of Mental Health; Brown University; Eli Lilly (Chair); BWH/HMS (Chair); University of Washington (Chair); Mayo Clinic, Rochester (Core PI) University of Southern California; UC San Diego; UC San Diego; UC San Diego; UC San Diego; UC San Diego; UC San Diego; UC San Diego; UC San Diego; UC San Diego; UC Davis (Core PI); UC Davis; UC San Diego; Mayo Clinic, Rochester (Core PI); Mayo Clinic, Rochester; University of London; UCLA School of Medicine; UCSF MRI; UC Davis; Mayo Clinic; Mayo Clinic; Mayo Clinic; Mayo Clinic; Mayo Clinic; Mayo Clinic; Mayo Clinic; UC Berkeley (Core PI); University of Michigan; University of Utah; Banner Alzheimer's Institute; Banner Alzheimer's Institute; University of Pittsburgh; UC Berkeley; Washington University St. Louis; Washington University St. Louis; Washington University St. Louis; Washington University St. Louis; UPenn School of Medicine; UPenn School of Medicine; UPenn School of Medicine; UPenn School of Medicine; UPenn School of Medicine; USC (Core PI); USC; USC; Indiana University; Indiana University; UC Irvine; Indiana University; Indiana University; Indiana University; Indiana University; UC San Francisco; UC San Diego; Prevent Alzheimer's Disease 2020; UC San Diego; National Institute on Aging; UC San Francisco; Brown University; National Institute of Mental Health; Cornell University; Johns Hopkins University; Richard Frank Consulting; Prevent Alzheimer's Disease 2020; National Institute on Aging; Oregon Health & Science University; University of Southern California; University of California - San Diego; University of Michigan; Mayo Clinic, Rochester; Baylor College of Medicine; Columbia University Medical Center; Washington University, St. Louis; University of Alabama - Birmingham; Mount Sinai School of Medicine; Rush University Medical Center; Wien Center; Johns Hopkins University; New York University; Duke University Medical Center; University of Pennsylvania; University of Kentucky; University of Pittsburgh; University of Rochester Medical Center; University of California, Irvine; University of Texas Southwestern Medical School; Emory University; University of Kansas, Medical Center; University of California, Los Angeles; Mayo Clinic, Jacksonville; Indiana University; Yale University School of Medicine; McGill Univ., Montreal-Jewish General Hospital; Sunnybrook Health Sciences, Ontario; U.B.C. Clinic for AD & Related Disorders; Cognitive Neurology - St. Joseph's, Ontario; Cleveland Clinic Lou Ruvo Center for Brain Health; Northwestern University; Premiere Research Inst (Palm Beach Neurology); Georgetown University Medical Center; Brigham and Women's Hospital; Stanford University; Banner Sun Health Research Institute; Boston University; Howard University; Case Western Reserve University; University of California, Davis - Sacramento; Neurological Care of CNY; Parkwood Hospital; University of Wisconsin; University of California, Irvine - BIC; Banner Alzheimer's Institute; Dent Neurologic Institute; Ohio State University; Albany Medical College; Hartford Hospital, Olin Neuropsychiatry Research Center; Dartmouth-Hitchcock Medical Center; Wake Forest University Health Sciences; Rhode Island Hospital; Butler Hospital; UC San Francisco; Medical University South Carolina; St. Joseph's Health Care; Nathan Kline Institute; University of Iowa College of Medicine; Cornell University; University of South Florida: USF Health Byrd Alzheimer's Institute; University of California, San Francisco; University of Southern California; UC San Francisco; University of Southern California; Mayo Clinic, Rochester; Brigham and Women's Hospital/ Harvard Medical School; UC Davis; Mayo Clinic, Rochester; UC Berkeley; Washington University St. Louis; Indiana University; Perelman School of Medicine, UPenn; USC; Perelman School of Medicine, University of Pennsylvania; UC San Francisco; Rehabilitation Institute of Chicago, Feinberg School of Medicine, Northwestern University; BWH/HMS (Chair); University of Washington (Chair); Core PI; Mayo Clinic, Rochester (Core PI); University of Southern California; UC San Diego; UC San Diego; UC San Diego; UC San Diego; UC San Diego; UC San Diego; UC San Diego; UC San Francisco; UC San Francisco; UC San Francisco; UC Davis (Core PI); UC San Diego; Mayo Clinic, Rochester (Core PI); Mayo Clinic, Rochester; Mayo Clinic; Mayo Clinic; Mayo Clinic; Mayo Clinic; Mayo Clinic; UC Berkeley (Core PI); University of Michigan; University of Utah; Banner Alzheimer's Institute; Banner Alzheimer's Institute; UC Berkeley; Washington University St. Louis; Washington University St. Louis; Washington University St. Louis; Perelman School of Medicine, UPenn; Perelman School of Medicine, UPenn; Perelman School of Medicine, UPenn; Perelman School of Medicine, UPenn; Perelman School of Medicine, UPenn; USC (Core PI); USC; USC; Indiana University; Indiana University; UC Irvine; Indiana University; Indiana University; Indiana University; Indiana University; UC San Francisco; Department of Defense (retired); University of Southern California; University of California, San Diego; Columbia University Medical Center; Rush University Medical Center; Wien Center; Duke University Medical Center; University of Rochester Medical Center; University of California, Irvine; Medical University South Carolina; Premiere Research Inst (Palm Beach Neurology); University of California, San Francisco; Georgetown University Medical Center; Brigham and Women's Hospital; Banner Sun Health Research Institute; Howard University; University of Wisconsin; University of Washington; Stanford University; Cornell University; ADNI Depression; Principal Investigator; University of California, San Francisco; ATRI PI and Director of Coordinating Center Clinical Core; University of Southern California; University of Southern California; Executive Committee; UC San Francisco; UC San Francisco; University of Southern California; University of Southern California; Mayo Clinic, Rochester; UC Berkeley; Indiana University; University of Southern California; UC Davis; University of Michigan; Data and Publication Committee (DPC); BWH/HMS (Chair); BWM/HMS (Director); Clinical Core Leaders; Core PI; University of Southern California; University of Southern California; University of Southern California; Clinical Informatics, Operations and Regulatory Affairs; USC; USC; USC; USC; USC; USC; USC; Psychiatry Site Leaders and Key Personnel; UC San Francisco; UC San Francisco; UC San Francisco; University of Pittsburgh; University of Pittsburgh; MRI Core Leaders and Key Personnel; Mayo Clinic, Rochester (Core PI); Mayo Clinic, Rochester; Mayo Clinic, Rochester; Mayo Clinic, Rochester; Mayo Clinic, Rochester; Mayo Clinic, Rochester; Mayo Clinic, Rochester; Mayo Clinic, Rochester; PET Core Leaders and Key Personnel; University of Michigan; UC Berkeley; Informatics Core Leaders and Key Personnel; USC (Core PI); USC; USC; Genetics Core Leaders and Key Personnel; Indiana University; Indiana University; Indiana University; Indiana University; Indiana University; University of California, San Francisco: University of Pittsburgh; ^1^Cleveland Clinic Lou Ruvo Center for Brain Health, Las Vegas, NV, United States; ^2^Departments of Psychology and Neuroscience, University of Colorado, Boulder, CO, United States

**Keywords:** sparse principal component analysis, PCA, canonical correlation analysis, CCA, data fusion, mild cognitive impairment, MCI

## Abstract

Collecting multiple modalities of neuroimaging data on the same subject is increasingly becoming the norm in clinical practice and research. Fusing multiple modalities to find related patterns is a challenge in neuroimaging analysis. Canonical correlation analysis (CCA) is commonly used as a symmetric data fusion technique to find related patterns among multiple modalities. In CCA-based data fusion, principal component analysis (PCA) is frequently applied as a preprocessing step to reduce data dimension followed by CCA on dimension-reduced data. PCA, however, does not differentiate between informative voxels from non-informative voxels in the dimension reduction step. Sparse PCA (sPCA) extends traditional PCA by adding sparse regularization that assigns zero weights to non-informative voxels. In this study, sPCA is incorporated into CCA-based fusion analysis and applied on neuroimaging data. A cross-validation method is developed and validated to optimize the parameters in sPCA. Different simulations are carried out to evaluate the improvement by introducing sparsity constraint to PCA. Four fusion methods including sPCA+CCA, PCA+CCA, parallel ICA and sparse CCA were applied on structural and functional magnetic resonance imaging data of mild cognitive impairment subjects and normal controls. Our results indicate that sPCA significantly can reduce the impact of non-informative voxels and lead to improved statistical power in uncovering disease-related patterns by a fusion analysis.

## Introduction

Collecting multiple modalities of neuroimaging data on the same subject is increasingly becoming the norm in clinical practice and research. Neuroimaging multi-modality data were traditionally analyzed and interpreted separately to find disease-related or task-related patterns in the brain. However, analyzing each modality independently does not necessarily find related patterns in both modalities. A single pattern in one modality might be related with a mixture of patterns in another modality. Fusing multiple modalities to find related patterns is a challenge in neuroimaging analysis. In the last decade, several techniques were proposed to utilize multiple imaging modalities, including data integration (Savopol and Armenakis, 2002; Calhoun and Adal, 2009), asymmetric data fusion (Filippi et al., 2001; Kim et al., 2003; Henson et al., 2010) and symmetric data fusion techniques (Correa et al., 2008; Groves et al., 2011; Sui et al., 2011; Le Floch et al., 2012; Lin et al., 2014; Mohammadi-Nejad et al., 2017). A detailed review about these techniques can be found in Calhoun and Sui (2016). In the data integration technique, each dataset is analyzed independently, and, then, one dataset is overlaid on another without considering the interaction among datasets. Asymmetric data fusion utilizes one dataset to improve the analysis of another dataset. For example, Kim et al. (2003) used the foci of functional magnetic resonance imaging (fMRI) activation as seed points for Diffusion Tensor Imaging fiber reconstruction algorithms. Filippi et al. (2001) integrated conventional magnetic resonance imaging (MRI) and diffusion tensor MRI to better locate white matter lesions in multiple sclerosis subjects. Henson et al. (2010) constrained the electromagnetic sources of Magnetoencephalography and Electroencephalography (MEG, EEG) data with fMRI as empirical priors. Along with advantages of asymmetric data fusion techniques, asymmetric fusion omits the fact that each imaging modality has an essentially unique nature (Calhoun and Sui, 2016). In the symmetric data fusion method, multiple imaging modalities are analyzed conjointly to optimize the information contributed by each modality. Multiple imaging modalities are combined to extract complementary information regarding the integrity of the underlying neural structures and networks (Calhoun and Sui, 2016). In this study, we focus on symmetric data fusion using two modalities. Unless explicitly stated, data fusion refers to symmetric data fusion.

Canonical correlation analysis (CCA) is a multivariate method of finding linear combinations of two multidimensional random variables to maximize their correlation (Hotelling, 1936). CCA and its extensions have been extensively utilized in data fusion to associate related patterns across multiple data. A few CCA-based fusion methods were proposed in the last decade, such as multimodal CCA (Correa et al., 2008), source CCA + joint ICA (Sui et al., 2010) and multimodal CCA + joint ICA (Sui et al., 2011). The variant of CCA with more than two datasets, multiset CCA, was also applied in data fusion (Correa et al., 2010). When CCA is directly applied to the original data in a fusion analysis, some of the canonical variables are perfectly correlated regardless of the association among data, since the feature space is usually high-dimensional and only relatively few observations (subjects) are available (Pezeshki et al., 2004). In the CCA-based fusion methods mentioned above, principal component analysis (PCA) was used to reduce the data dimension. More specifically, a set of principal components with the largest possible variances are found by PCA and then the projections of original data (scores) on the space spanned by principal components are the dimension-reduced input data for the fusion CCA algorithm.

PCA solves the singularity problem in these fusion methods but does not take into account that in many cases only a small proportion of voxels (features), called informative voxels (features), have contribution to the variance, and a large proportion are non-informative. If principal components were obtained with non-informative voxels (features) assigned to zero, the projections of original data on the space spanned by the major principal components are more robust to non-informative voxels and thus helps CCA to better match related patterns across modalities. For example, when fusion analysis is applied to the data acquired from mild cognitive impairment (MCI) subjects and normal controls (NC), brain regions engaged in memory, language, and judgment (e.g., hippocampus, medial temporal lobe, frontal lobe) should be significant in the disease-related patterns (Forsberg et al., 2008; Bai et al., 2009). Specifying non-informative voxels to have zero weight could be beneficial for matching disease-related patterns by a fusion analysis. In general, properly suppressing non-informative voxels will further improve the statistical power of fusion techniques. Even though imaging data can be masked with predetermined regions of interest (ROIs) to address the feature selection process and avoid problems arising from non-informative voxels, ROI selection requires typically unavailable prior knowledge about the disease and patient cohort.

Selection and suppression of non-informative features in principal components can be automated by implementing sparsity in the PCA algorithm, called *sparse* PCA (sPCA) (Zou et al., 2006; Witten et al., 2009). The sPCA method and its extensions have been applied in multiple fields, such as machine learning, pattern recognition, and bioinformatics (Zou et al., 2006; Shen and Huang, 2008; Witten et al., 2009; Jenatton et al., 2010). A brief review of sPCA can be found in Feng et al. (2016). When comparing sPCA+CCA with PCA+CCA, sPCA produces different scores because of the reoriented space spanned by the principal components and, thus, sPCA influences the subsequent CCA step in associating multiple modalities.

Unlike sPCA+CCA having feature selection prior to fusing datasets, sparse CCA (sCCA) (Parkhomenko et al., 2009; Witten and Tibshirani, 2009; Lê Cao et al., 2011; Abdel-Rahman et al., 2014; Avants et al., 2014) has feature selection and data fusion applied at the same time. In this study, the sPCA+CCA method is compared with the sCCA method.

In the following, we first describe the theory behind sPCA and outline how to implement the sPCA algorithm. Then, we develop a cross-validation algorithm to optimally specify the sparsity parameter and the number of major principal components in sPCA. Then, we evaluate the improvement by introducing sparsity constraint to PCA using simulated data. Considering mild cognitive impairment (MCI) impacts both the function and structure in certain regions of the brain (Chetelat et al., 2002; Rombouts et al., 2005), we apply four fusion methods including sPCA+CCA, PCA+CCA [called multimodal CCA in Correa et al. (2008)], sCCA (Witten et al., 2009) and parallel ICA (Liu et al., 2009) on structural and functional MRI data of mild cognitive impairment (MCI) subjects and normal controls (NC), with the hypothesis to find disease-related association between these two modalities. Since disease-related features are visible in all modalities to varying degrees (Groves et al., 2011), fusion methods can match disease-related patterns in a two-group setting. Hence, the group discrimination and the correlation with β-amyloid measurement can be used to evaluate how well fusion methods match disease-related patterns across modalities.

## Theory

### Sparse Principal Component Analysis (sPCA)

#### Derivation of sPCA

Let ***X*** denote an *n* × *m* feature matrix with rank(***X***) ≤ min(*n, m*), where *n* is the number of observations and *m* is the number of features in each observation. If ***X*** is a brain map, as in our case, *n* is the number of subjects and *m* is the number of voxels. PCA transforms a set of observations of correlated variables into a set of uncorrelated orthogonal variables called principal components that can be ordered according to the magnitude of their eigenvalues. The first *K* principal components can be determined by minimizing the least square problem (Eckart and Young, 1936), expressed as

(1) $$f_{obj}=\underset{\hat{X}\in M(K)}{\min}\frac{1}{2}||X-\hat{X}|{|}_{F}^{2},$$

where *M*(*K*) is a set of matrices with rank(*M*) = *K* and ${\left\|.\right\|}_{F}^{2}$ means the squared Frobenius norm (see Appendix A in Supplementary Material for more detail). PCA is closely related to singular value decomposition (SVD). Using SVD, ***X*** can be decomposed into

(2) $$X=UDV^{T},\;U^{T}U=I_{K},\;V^{T}V=I_{K},$$

where ***U*** ∈ ℝ^*n*×*K*^ *and* ***V*** ∈ ℝ^*m*×*K*^ are the left and right singular vectors of ***X*** satisfying the orthonormality condition, and ***D* =***diag***(***d*_1_, …, *d*_*K*_) ∈ ℝ^*K*×*K*^is the diagonal matrix of ordered singular values of ***X*** with *d*_1_ ≥ *d*_2_ ≥ … ≥*d*_*K*_ > 0. The optimal $\hat{X}$ in *M*(*K*) can be written as

(3) $$\hat{X}=\sum_{i=1}^{K}d_{i}u_{i}v_{i}^{T},$$

where $u_{i}\in {\mathbb{R}}^{n\times 1}$ and $v_{i}\in {\mathbb{R}}^{m\times 1}$ denote the *i*-th column vector of ***U*** and ***V***, respectively. Following the notation in SVD, the objective function *f*_*obj*_ for only one component can be written as

(4) $$f_{obj}\left(d,u,v\right)=\frac{1}{2}{\left\|X-duv^{T}\right\|}_{F}^{2},\;s.t.\;{\left\|u\right\|}_{2}^{2}=1,\;{\left\|v\right\|}_{2}^{2}=1,\;d>0$$

Considering that there are many voxels but few subjects, namely, *m* ≫ *n*, the sparsity in our study is only implemented to set non-informative voxels to be zero. Because ***V*** is a set of voxel-wise spatial maps, sparsity was incorporated into the projection vector ***v***_*i*_ but not the score vector **u**_*i*_, which is different than the sPCA method in Witten et al. (2009), who applied sparsity constraint on both singular vectors ***u*** and ***v***. For this reason, we derived the sPCA formula with an *L*_1_ penalty on variable **v** added to the *f*_*obj*_(*d*, **u**, **v**) in Equation (4):

(5) $$f_{obj}\left(d,u,v\right)=\frac{1}{2}{\left\|X-duv^{T}\right\|}_{F}^{2},\;s.t.\;{\left\|u\right\|}_{2}^{2}=1,\;{\left\|v\right\|}_{2}^{2}=1,\;{\left\|v\right\|}_{1}\leq c,\;d>0,$$

where the parameter *c* is the *sparsity tuning parameter*. A smaller *c* means that more elements in the principal component ***v*** are set to zero and the principal component becomes sparser. We would like to emphasize that sPCA has the elastic penalty consisting of the *L*_1_ and *L*_2_ penalty as shown in Appendix B in Supplementary Material, and, thus, the principal components from sPCA are well-defined and unique even when *m* ≫ *n* (Zou et al., 2006). Following the derivation in Appendix A in Supplementary Material, Equation (5) can be rewritten as:

(6) $$\underset{u,v}{\text{maximize}}d=u^{T}Xv,\;s.t.\;{\left\|u\right\|}_{2}^{2}=1,\;{\left\|v\right\|}_{2}^{2}=1,\;{\left\|v\right\|}_{1}\leq c.$$

As shown by Witten et al. (2009), if ***u*** or ***v*** is fixed, the criterion in Equation (6) is a convex problem in ***v*** or ***u***.Thus, Equation (6) represents a biconvex problem. Because a convex problem can be solved reliably and efficiently, we solve Equation (6) by converting the equation into two convex sub-problems with **u** and **v** *alternatingly* fixed.

#### Iterative Algorithm for sPCA

Equation (6) is solved by an iterative algorithm modified based on the sPCA algorithm in Witten et al. (2009). We start with an initial value $u=\frac{u}{||u|{|}_{2}}$ and then update **v** to maximize ***u***^*T*^***Xv*** as expressed below

(7) $$\underset{v}{\text{maximize}}a^{T}v\;s.t.\;{\left\|v\right\|}_{2}^{2}=1,\;{\left\|v\right\|}_{1}\leq c,\;a=X^{T}u.$$

Appendix C in Supplementary Material shows that the optimal solution in Equation (7) is $v=\frac{S\left(a,\mu \right)}{||S\left(a,\mu \right)|{|}_{2}}$. The function *S* is the (vector-valued) soft threshold function given by *S*(***a***, μ) = *sign*(***a***)max(0, |***a***|−μ), where the *sign* (**.**) and |**.**| operation act on each element of vector ***a***. If μ = 0 satisfies ||***v***||_1_ ≤ *c*, then $v=\frac{a}{||a|{|}_{2}}$. Otherwise, μ is determined efficiently by a binary search algorithm to have ||***v***||_1_ = *c*. At a fixed ***v***, Equation (6) becomes

(8) $$\underset{u}{\text{maximize}}u^{T}b\;s.t.\;{\left\|u\right\|}_{2}^{2}=1,\;b=X^{T}v.$$

The optimal ***u*** is simply the unit vector along direction ***b***, namely, $u=\frac{b}{||b|{|}_{2}}=\frac{Xv}{||Xv|{|}_{2}}$. The alternating iteration stops when a convergence criterion is satisfied. Then ***X*** is updated by removing the variance contained in the previous principal component by ***X*←*X*−***d****uv***^*T*^, and the next pair of ***u*** and ***v*** is computed by the same iterative algorithm until *K* principal components are found.

#### Parameters Selection in sPCA by Split-Sample Cross Validation

**Figure 1:**
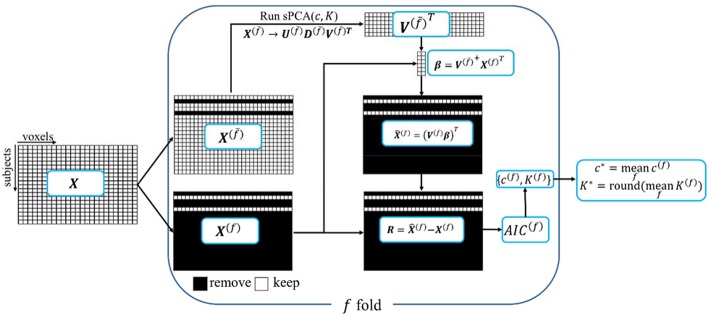
A schematic diagram of the split-sample cross-validation method in sPCA. ***X*** is the data matrix. Each subject is randomly assigned to one fold, ***X***^(*f*)^ denotes the data from the subjects assigned in ***f***-fold data and ***X***^(*f*)^ denotes the data with ***X***^(*f*)^ excluded. The “+” superscript indicates the Moore-Penrose pseudoinverse.

A ten-fold cross validation method is used to estimate the parameters in sPCA, including the optimal sparsity tuning parameter c^*^ and the best number of principal components K^*^. The flow chart for the *split-sample cross validation method* is shown in Figure 1. For data matrix ***X***, each subject is randomly assigned to one fold. Let ***X***^**(***f***)**^ denote the data from the subjects assigned in the *f*-fold dataset and $X^{\left(\bar{f}\right)}$ denote the data except the data in the *f*-fold dataset. Principal components are computed from matrix $X^{\left(\bar{f}\right)}$, and then these principal components are applied on ***X***^**(***f***)**^ to estimate parameters based on a selection criterion, and, finally, the mean value of the estimated parameters in each fold of the data is used for fusion analysis. Mathematically, *K*-factor sPCA is applied on matrix $X^{\left(\bar{f}\right)}$ by $X^{\left(\bar{f}\right)}\to \;U^{\left(\bar{f}\right)}D^{\left(\bar{f}\right)}V^{{\left(\bar{f}\right)}^{T}}$ where $U^{\left(\bar{f}\right)}=[u_{1}^{\left(\bar{f}\right)},\ldots ,\;u_{K}^{\left(\bar{f}\right)}],\;V^{\left(\bar{f}\right)}=[v_{1}^{\left(\bar{f}\right)},\ldots ,v_{K}^{\left(\bar{f}\right)}]\;$ and $D^{\left(\bar{f}\right)}=diag\left(\;d_{1}^{\left(\bar{f}\right)},\ldots ,\;d_{K}^{\left(\bar{f}\right)}\right)$. Then, the principal components $V^{\left(\bar{f}\right)}$ are used as regressors in a linear regression model to fit each sample in the untouched data ***X***^(*f*)^, namely, $V^{{\left(\bar{f}\right)}^{+}}X^{{\left(f\right)}^{T}}$ and ${\hat{X}}^{\left(f\right)}={\left(V^{\left(\bar{f}\right)}\;\beta \right)}^{T}$. The Akaike Information Criterion (*AIC*) (Akaike, 1974; Shumway et al., 2000) is used to evaluate how close the reconstructed matrix ${\hat{X}}^{\left(f\right)}$ is to ***X***^**(***f***)**^. The *AIC* provides a tradeoff between goodness-of-fit (minimum log-likelihood) and complexity of the model (Sui et al., 2010). Witten et al. (2009) used the mean-square-error (MSE) as the criterion in a cross-validation method that is based on an imputation algorithm (Troyanskaya et al., 2001). The optimal sparsity tuning parameter c^*^ was selected by minimizing MSE with only the first principal component (*K* = 1) considered. This method cannot estimate the number of principal components K^*^ since MSE always decreases with increasing *K*. We have revised the cross-validation method in Witten et al. (2009) with *AIC* as the criterion and compared *AIC* with the split-sample cross-validation method. We found that the split-sample method is more reliable and accurate in estimating parameters. Appendix D in Supplementary Material describes the calculation of *AIC* and the comparison of these two cross-validation methods in more detail. Let {*c*^(*f*)^, *K*^(*f*)^} denote the parameters having minimum *AIC* for the *f*-fold cross-validation, the optimal sparsity tuning parameter c^*^ is defined as the average over *c*^(*f*)^, and the optimal number of principal components K^*^ is the rounded integer of the average over *K*^(*f*)^. The estimated parameter set {c^*^, K^*^} is used in the sPCA+CCA fusion analysis.

### sPCA+CCA

In sPCA+CCA, PCA is replaced by sPCA for dimension-reduction. The sPCA method is applied to reduce the data dimension for each modality separately, i.e., $X_{r}\to {\hat{X}}_{r}=U_{r}D_{r}V_{r}^{T},\;r=1,2$. In this step, the sparsity tuning parameter $c_{r}^{*}$, r = 1,2, and the number of principal components $K_{r}^{*}$, r = 1,2, are optimized for each modality by using the split-sample cross-validation method described in section Parameters Selection in sPCA by Split-Sample Cross Validation. The dimension-reduced dataset $Y_{r}\in R^{n\times K_{r}^{*}}$ is the principal component score given by

(9) $$Y_{r}={\hat{X}}_{r}V_{r},\;r=1,2.$$

**Figure 2:**
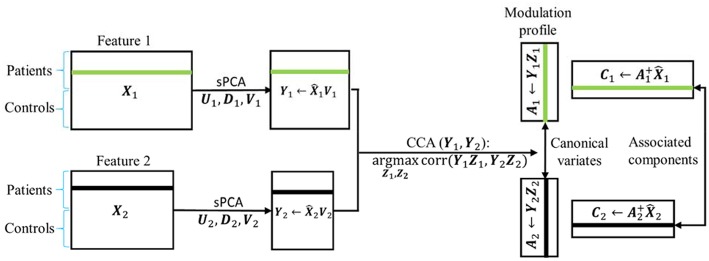
Flow chart of sPCA+CCA. sPCA was carried out reduce data dimensions and to suppress irrelevant features. Then, CCA was carried out for fusion analysis to obtain modulation profiles and associated components. In the flow chart ${\hat{X}}_{r}=U_{r}D_{r}V_{r}^{T}\;with\;r=1,2$, is the data matrix of the two modalities obtained from sPCA.

Then, CCA is applied to link the data ***Y***_1_ and ***Y***_2_ by maximizing the canonical correlation between ***Y***_1_***Z***_1_ and ***Y***_2_***Z***_2_, where ***Z***_*r*_, *r* = 1, 2, denote the canonical transformation matrices. The resulting canonical variates ***A***_*r*_ = ***Y***_*r*_***Z***_*r*_ are called modulation profiles. Only the matched modulation profiles between datasets are correlated, and all other modulation profiles are uncorrelated, i.e.,

(10) $$\begin{array}{l} A_{1d}^{T}A_{2d}=\rho_{d}>0,\;for\;d=1,\;\ldots ,\;D;D=\text{min}(K_{1}^{*},K_{2}^{*})\\ \;A_{r_{1}d_{1}}^{T}A_{r_{2}d_{2}}=0,\text{ for }d_{1}\neq d_{2}\text{ and }r_{1},r_{2}\in \left\{1,2\;\right\}, \end{array}$$

where ρ_*d*_ is the canonical correlation between ***A***_1*d*_ and ***A***_2*d*_. Finally, the spatial maps ***C***_1_ and ***C***_2_ corresponding to ***A***_1_ and ***A***_2_, respectively, are calculated by least square estimation according to

(11) $$C_{r}=A_{r}^{+}{\hat{X}}_{r},\;r=1,2$$

where the “+” superscript indicates the Moore-Penrose pseudoinverse. In Equations (9) and (11) we could have used the original data matrix ***X***_*r*_ instead of ${\hat{X}}_{r}$. A schematic flowchart of sPCA+CCA is shown in Figure 2.

## Materials and Methods

### Simulation 1: sPCA vs. PCA

**Figure 3:**
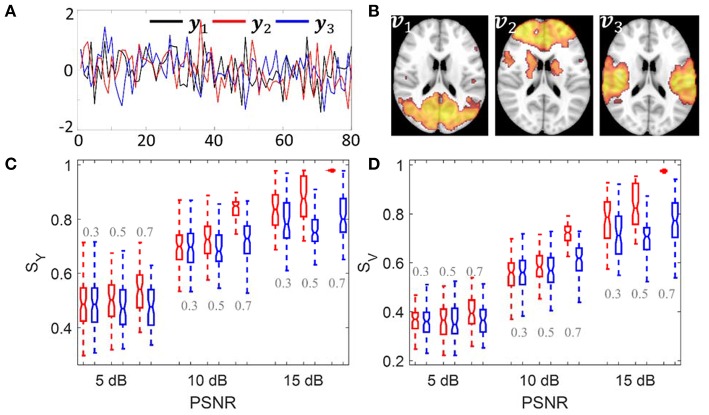
Simulation 1: Comparison of sPCA and PCA. **(A)** Simulated principal component scores ***Y*** = [***y***_1_, ***y***_2_, ***y***_3_]. **(B)** The spatial maps ***V*** = [***v***_1_, ***v***_2_, ***v***_3_] corresponding to the scores with 70% voxels having zero values. Simulated data were generated 100 times with PSNR as 5 dB, 10 dB and 15 dB and sparsity level as 0.3, 0.5, and 0.7. **(C)** Boxplot for the similarity value with true principal component scores ***S***_*Y*_. **(D)** Boxplot for the similarity value with true spatial maps ***S***_*V*_. The boxplot for sPCA is shown in red and for PCA in blue.

The simulation was carried out to evaluate whether sPCA is sensitive to the noise in the data at different sparsity levels. The simulated data ***X*** was generated based on the form ***X*** = ***YV***^*T*^, where ***Y* = […,*y***_**n**_**, …]** is the intrinsic principal component scores and ***V* = […,*v***_***n***_**, …]** is a set of orthogonal maps. The simulation consists of 80 samples and 3 intrinsic principal components, hence ***Y*** has a dimension of 80 ×3. To analyze whether the improvement made by introducing sparsity to PCA relates to the spatial sparsity level of the signal, we have simulated the data with sparsity levels of 30, 50, and 70%. Here, the sparsity level is defined as the percentage of zero elements in the map. Figure 3 shows the principal component scores ***Y*** in Figure 3A and their corresponding spatial maps ***V*** at 70% sparsity level in Figure 3B without threshold. The images have a dimension of 91 ×109 ×3, and only the second slice of the spatial maps is shown.

Gaussian noise ***N*** was added to create noisy images and Gaussian smoothing with Full-Width-At-Half-Maximum (FWHM) of 8 mm was applied to introduce spatial correlation. The simulated data were generated with Peak Signal-to-Noise Ratio (PSNR) of 5, 10, and 15 dB, which are similar to the PSNRs used in Sui et al. (2010). PSNR is defined as

(12) $$PSNR=20\;log_{10}\frac{maxval}{MSE}.$$

Here, *maxval* is the maximum possible pixel value and MSE is the mean squared error between noisy and noise-free images. A higher PSNR indicates a higher image quality. The simulation was carried out 100 times using the same ***Y*** and ***V***, but with different noise realizations.

### Simulation 2: Comparison of Fusion Methods

**Figure 4:**
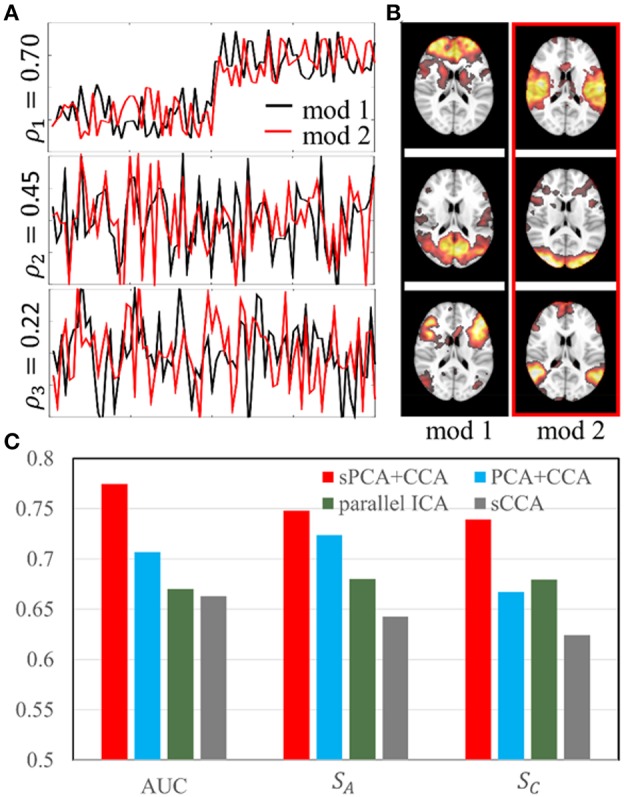
Simulation 2: Comparison of fusion methods. **(A)**. Three pairs of simulated modulation profiles satisfying the orthogonality condition. **(B)**. Simulated spatial maps at sparsity level 70%. **(C)** Bar plot of the **AUC** measurement, similarities (**S**_*A*_) between estimated and true modulation profiles, and their corresponding spatial maps (**S**_*C*_).

The simulation was carried out with sparsity level 70% at moderate signal-to-noise ratio with PSNR = 10 dB. The sparsity level used in the simulation is close to the estimated sparsity level in the real data as mentioned below in Parameter Selection section. Two simulated modalities were generated by following the steps described in section Simulation 1: sPCA vs. PCA except we replace the intrinsic principal component scores by modulation profile ***A***_1_ and ***A***_2_, respectively, for the first and second modality. The modulation profiles {***A***_1_, ***A***_2_} satisfy the orthogonality condition in Equation (10). The canonical correlations ρ between ***A***_1_ (red curve) and ***A***_2_ (black curve) are [0.70, 0.45, 0.22] as shown in Figure 4A. The three corresponding pairs of sparse spatial maps are shown in Figure 4B. The first pair of canonical variables in {***A***_1_, ***A***_2_} were simulated to be group-distinct using 40 subjects for each group. The simulation was carried out fifty times using the same modulation profiles and spatial maps, but with different noise realizations. The average performance is reported in the Result section.

### MRI/fMRI Data and PET Analysis

Structural MRI and resting-state fMRI data used in this study were downloaded from the publicly available ADNI database. The ADNI was launched in 2003 as a public-private partnership, led by Principal Investigator Michael W. Weiner, MD. The primary goal of ADNI has been to test whether serial MRI, positron emission tomography (PET), other biological markers, and clinical and neuropsychological assessment can be combined to measure the progression of mild cognitive impairment (MCI) and early Alzheimer's disease (AD).

The resting-state fMRI data, T1 structural data, and corresponding clinical data were downloaded from the ADNI 2 database before September 18, 2016. All subjects used in this study had florbetapir (^18^F) PET scans within 6 months of MRI scans. All MCI subjects had an absence of dementia (clinical dementia rating of 0.5), a memory complaint and objective memory loss measured by education adjusted scores on the Wechsler Logical Memory Scale II, an absence of significant levels of impairment in other cognitive domains and essentially had preserved activities of daily living. All subjects were scanned on a 3.0-Tesla Philips MRI scanner. The magnetization prepared rapid acquisition gradient echo (MP-RAGE) sequence was used to acquire T1-weighted structural images by the investigators of the ADNI consortium. The structural MRI scans were collected with a 24 cm field of view and a resolution of 256 ×256 ×170, to yield a voxel size of 1 ×1 ×1.2 mm. Resting-state fMRI data were acquired from an echo-planar imaging sequence with parameters: 140 time points; TR/TE = 3000/30 ms; flip angle = 80 degrees; 48 slices; spatial resolution = 3.3 mm ×3.3 mm ×3.3 mm and imaging matrix = 64 ×64. Details of the ADNI MRI protocol can be found on ADNI website (http://adni.loni.usc.edu/). If one subject had multiple MRI/fMRI scans satisfying the requirements specified above, the first available MRI/fMRI data set was used for analysis. The Standard Uptake Value Ratio (SUVR) analysis was carried out to measure ß-amyloid on ADNI florbetapir PET scans by site investigators and the SUVR data using a composite reference regions were downloaded from the ADNI website. The correlation between SUVR measurement and the result of fusion methods was used to evaluate the performance of different fusion methods. In total, 37 MCI subjects (age = 73.7 ± 6.7 years; gender = 19 female/18 male) and 42 NC subjects (age = 75.0 ± 7.3 years; gender = 24 female/18 male) were selected.

### FMRI Data

#### Preprocessing

The first 5 volumes were excluded from the analysis. The fMRI time series were slice-timing corrected and realigned to the first volume using SPM12, co-registered to the individual T1 images and then normalized to the MNI152 2 mm template using Advanced Normalization Tools (ANTs) (http://stnava.github.io/ANTs/). Nuisance regression was carried out with six head motion parameters along with signals extracted from white matter and CSF [3-mm radius spheres centered at MNI coordinates (26, −12, 35) and (19, −33, 18)] (Chen et al., 2015). The resulting time series were smoothed further with a 10-mm Gaussian kernel and band-pass filtered to be in the frequency range 0.01–0.1 Hz. These steps were computed with MATLAB (The Mathworks, Inc., version R2015a).

#### Eigenvector Centrality Mapping (ECM)

Many studies have shown that graph-theoretical analysis methods can help elucidate the disruption of brain network structure in patients compared to normal controls (He and Evans, 2010; Power et al., 2011). In graph theory and network analysis, centrality is a measure of importance of a node in the graph (Bavelas, 1948). We used eigenvector centrality mapping (ECM) to analyze functional networks. ECM is an assumption-free non-parametric method that can efficiently carry out voxel-wise whole brain nodal analysis. A variant of eigenvector centrality that has been applied successfully is Google's PageRank algorithm (Bryan and Leise, 2006), which is used as the Google search engine.

In the ECM algorithm, a *m* × *m* similarity matrix (for example a correlation map between voxel-wise time series) is constructed and the eigenvector centrality map is the eigenvector corresponding to the largest eigenvalue of the similarity matrix. Here, the value at node (voxel) *i* is defined as the *i*-th entry in the normalized eigenvector. Because the normalization step in ECM reduces the centrality value in a map with more nodes, a group mask with the same nodes is used for all subjects when applying ECM on fMRI data. Individual masks were first calculated by thresholding the mean fMRI signal intensity at 10% of the maximal mean signal intensity for each individual subject, and then the group mask was chosen to be the intersection of all individual masks and the MNI152 gray matter mask. The ECM maps of resting-state fMRI time series for all subjects were calculated using the Fast ECM algorithm (Wink et al., 2012). Unlike the basic ECM algorithm, the Fast ECM algorithm can estimate voxel-wise eigenvector centralities computationally more efficiently because the Fast ECM computes matrix-vector products directly from the data without explicitly storing the correlation matrix. The 3D ECM map of each subject was masked and reshaped to a one-dimensional vector with 128257 non-zero voxels and the ECM maps of all subjects were represented as a two-dimensional array of dimension 79 ×128257. The ECM maps were corrected by regressing out the effects of age, gender and handedness. The corrected ECM matrix, denoted as **X**_*ECM*_, was used for fusion analysis.

### T1 Images

#### Voxel-Based Morphometry (VBM)

VBM is a common automated brain segmentation technique that is used to investigate structural brain difference (volume differences) among different populations (Ashburner and Friston, 2000). A standard VBM processing routine was created with the SPM12-DARTEL toolbox (Ashburner, 2007). The following processing steps were carried out for VBM: (a) the raw T1 structural images were bias-corrected for inhomogeneities, brain-extracted and segmented (“Native+DARTEL imported” is selected in “Native Tissue” option) into gray mater, white mater and cerebrospinal fluid probability maps; (b) a customized template was created using the SPM12-DARTEL “create template” module; (c) gray mater volumes for all subjects were normalized and registered to the MNI152 2 mm template using the final DARTEL template in “create template” module and finally smoothed using an 8 mm FWHM Gaussian filter. The 3D VBM map of each subject was masked and reshaped to a one-dimensional vector of 171705 non-zero voxels and the VBM maps of all subjects were represented as a two-dimensional array of dimension 79 x 171705. The VBM maps were corrected by regressing out the effects of age, gender and handedness. The corrected VBM matrix, denoted as **X**_*VBM*_, constitute the other modality used for fusion analysis.

### sPCA+CCA, PCA+CCA, sCCA, and Parallel ICA

To see the improvement achieved by replacing PCA with sPCA, both sPCA+CCA and PCA+CCA were performed on simulated and real imaging data. In addition, sPCA+CCA was also compared with parallel ICA using the Fusion ICA Toolbox (FIT, http://mialab.mrn.org/software/fit/). Furthermore, a comparison with *sparse* CCA (sCCA) was carried out.

#### Parallel ICA

Similar to ICA computing maximally independent components in one dataset, parallel ICA finds the hidden independent components from two datasets simultaneously with the association between modalities considered (Liu et al., 2009). Parallel ICA is realized by jointly maximizing the independence among components in each modality and the correlations between modalities in a *single* algorithm. The maximal number of correlated components *n*_*cc*_ are pre-defined and only the correlation above threshold ρ_*thre*_ is considered. More detailed description can be found in Liu et al. (2009) and the fusion ICA toolbox (Fulop and Fitz, 2006) (http://mialab.mrn.org/software/fit/) documentation. Parallel ICA was carried out with standard PCA and the default “AA” parallel ICA algorithm using the FIT toolbox. Parallel ICA was repeated ten times for consistency. The default ICA options were used in the analysis. The maximally allowed descending trend of entropy was −0.001, the maximum number of steps was 512 and the default learning rates (0.0063, 0.0065) were used. Since the performance of parallel ICA depends on the hyperparameters including the maximal number of correlated components *n*_*cc*_ and the correlation threshold ρ_*thre*_, we have used five pairs of hyperparameters, {*n*_*cc*_, ρ_*thre*_} = {1, 0.2}, {1, 0.4}, {3, 0.3}, {5, 0.2}, and {5, 0.4} for both simulated and real data. The best performance was used to compare with other fusion methods.

#### Sparse CCA (sCCA)

Unlike the sPCA+CCA method that enforces sparsity during the dimension reduction step, sCCA associates the original data ***X***_1_ and ***X***_2_ directly with a *sparsity constraint applied on the canonical transformation matrices*. The obtained transformation matrices and the canonical variates present the spatial maps ***C***_*r*_ and the modulation profiles ***A***_*r*_, respectively. The iterative algorithm for sCCA is described in detail in Witten et al. (2009).

#### Parameter Selection

To avoid overfitting, we carried out parameter selection for all fusion methods. The number of sparse principal components used in sPCA+CCA was determined by the AIC-based *split-sample cross-validation method* described in section Parameters Selection in sPCA by Split-Sample Cross Validation. For the ECM and VBM modalities, the optimal numbers of principal components were 10 and 7, respectively, and the optimal sparsity levels were 70 and 80%, respectively. The same cross-validation method was also used to determine the number of conventional principal components in PCA+CCA and in parallel ICA by simply replacing sPCA with the standard PCA algorithm. 7 ECM principal components and 6 VBM principal components were found for these two fusion methods. While minimizing MSE based on CCA potentially can be used to select the parameters in sPCA+CCA or PCA+CCA as suggested by Lameiro and Schreier (2016), the high dimensionality of the data and the SVD over a large cross-covariance matrix makes this parameter selection method infeasible for our study because of computational time and memory. The optimal sparsity tuning parameter in sCCA was estimated by the cross-validation method presented in Witten and Tibshirani (2009). With this method, the optimal sparsity level is 73% for the ECM dataset and 79% for the VBM dataset.

To compare how well the sPCA and PCA methods extract the intrinsic principal component scores ***Y***^*true*^ and the spatial maps ***V***^*true*^ in simulation 1, the similarity between the estimated and the true scores and maps were computed at different noise levels by following equation

(13) $$\begin{array}{l} S_{Y}=\frac{\sum_{d=1}^{3}\left|corr\left(y_{d}^{true},\;y_{d}^{est}\right)\right|}{3}\in [0,1],\\ S_{V}=\frac{\sum_{d=1}^{3}\left|corr\left(v_{d}^{true},\;v_{d}^{est}\right)\right|}{3}\in [0,1]. \end{array}$$

The similarity value *S*_*Y*_ close to 1 indicates that the estimated ***Y***^*est*^ agrees well with the true scores ***Y***^*true*^. Similarly, the similarity value *S*_*V*_ close to 1 indicates that the estimated ***V***^*est*^ agrees well with the true spatial maps ***V***^*true*^.

When comparing the fusion methods in simulation 2, the evaluation is focused on how well these methods distinguish two groups and uncover the modulation profiles and their corresponding spatial maps. The receiver operating characteristic (ROC) was used to evaluate group classification and the area under ROC curves (AUC) were calculated. The similarity between true modulation profiles $A_{r}^{true}$ and the estimated one, $A_{r}^{est}$, was computed, namely,

(14) $$\begin{array}{l} S_{A}=\frac{\sum_{r=1}^{2}\sum_{d=1}^{3}\left|corr\left(A_{rd}^{true},A_{rd}^{est}\right)\right|}{6}\in [0,1],\\ S_{C}=\frac{\sum_{r=1}^{2}\sum_{d=1}^{3}\left|corr\left(C_{rd}^{true},C_{rd}^{est}\right)\right|}{6}\in [0,1]. \end{array}$$

The similarity for spatial maps ${\text{C}}_{r}^{est}$ as defined in Equation (11) was also computed. Furthermore, we used the correlation error $\rho =\overset{3}{\underset{d=1}{\sum }}\left(\rho_{d}^{true}-\rho_{d}^{est}\right)$ to measure how close the estimated correlation **ρ**^*est*^ and intrinsic (true) correlation **ρ**^*true*^ are. A positive sign of Δρ indicates that overall the correlation is underestimated and a negative sign indicates the correlation is overestimated.

For the real imaging data, the ECM array ***X***_*ECM*_ and VBM array ***X***_*VBM*_ were used for fusion analysis. Two sample *t*-tests with unequal variances were applied on modulation profiles ***A***_*ECM*_ and ***A***_*VBM*_. ROC analysis was carried out on modulation profiles ***A***_*ECM*_ and ***A***_*VBM*_ to determine how well fusion methods extract disease-related modulation profiles and corresponding patterns, and the *AUC* for each modality also was calculated.

## Results

### Simulations

Conventional PCA and sPCA were carried out 100 times on a series of simulated data with PSNR as 5 dB, 10 and 15 dB and the sparsity level as 30, 50, and 70%. The boxplot for similarity values *S*_*Y*_ and *S*_*V*_ of sPCA (red color) and PCA (blue color) were shown in Figure 3C and Figure 3D. When the sparsity level and PSNR is low (e.g., sparsity level = 30% and PSNR = 5 dB), the improvement by introducing sparsity constraint is negligible. With increasing PSNR or sparsity level, sPCA outperforms PCA in uncovering the true principal component scores and corresponding spatial maps.

In simulation 2, fusion analysis was carried out fifty times on the simulated data with PSNR 10 dB and sparsity level 70%. Figure 4C shows the mean value of AUC, *S*_*A*_ and *S*_*C*_ for these four fusion methods including sPCA+CCA, PCA+CCA, parallel ICA and sCCA. The sPCA+CCA has the best performance among these fusion methods. Compared to PCA+CCA, sPCA+CCA has improved measurements of AUC and *S*_*C*_ by approximately 10%. The correlation error Δρ for sPCA+CCA, PCA+CCA, parallel ICA and sCCA are 0.11, 0.16, 0.17, and −0.35, respectively. Results indicate that sPCA+CCA achieves correlations closest to the simulated correlations, and sCCA significantly overestimates the correlation while all other fusion methods underestimate the correlation.

### Real fMRI Data

#### sPCA vs. PCA

**Figure 5:**
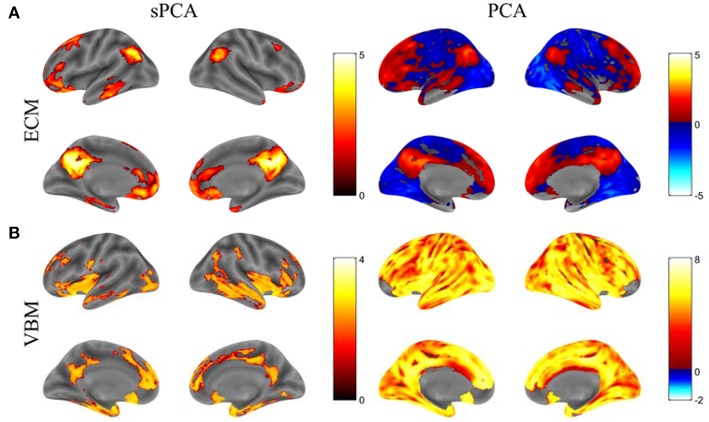
The standardized principal component maps having largest variance from sPCA and PCA. **(A)** ECM principal component, **(B)** VBM principal component.

The principal components having largest variance from sPCA and PCA are shown in Figure 5 without threshold for the ECM maps (Figure 5A) and VBM maps (Figure 5B). The color bars are different for these spatial maps to better visually represent the principal components. The ECM principal component obtained from sPCA shows a clear default mode network (DMN) pattern and the VBM principal component have non-zero voxels centered at the hippocampus. Compared to the spatial maps of PCA, sPCA has similar principal component maps but with a large proportion of voxels removed. A group comparison of the principal component scores from sPCA and PCA on ECM and VBM modality is applied. The sPCA method has achieved the most significant group difference with uncorrected *p*-value 0.01 and 0.008 on ECM and VBM modality, respectively. In contrast, PCA has obtained less significant group difference with uncorrected p-value 0.03 and 0.02 on ECM and VBM modality, respectively.

#### Fusion Analysis

For each fusion method, the modulation profiles ***A***_*ECM*_ and ***A***_*VBM*_ were calculated and two-sample *t*-tests with unequal variance were carried out to assess group difference. The ROC technique was applied on modulation profiles ***A***_*ECM*_ and ***A***_*VBM*_, and the AUC was calculated. Group classification accuracy was also calculated by running ten-fold quadratic discriminant analysis (QDA) on modulation profiles from both ECM and VBM modalities. The most significant component of ***A***_*ECM*_ or ***A***_*VBM*_ from two-sample *t*-tests always had the largest AUC value. The AUC and Bonferroni-corrected *p* value for multiple comparisons, denoted as *p*_*corr*_, of the most significant components are shown in Table 1. The correlation between the most significant components is also listed in this table. sPCA+CCA found one significant component in both ECM and VBM data (ECM: $p_{corr}=3.4\times 10^{-4},\;AUC=0.78$; VBM: $p_{corr}=2.6\times 10^{-4},\;AUC=0.81$). sPCA+CCA associated these two significant components at the 1st pair of canonical variates with canonical correlation ρ = 0.78. PCA+CCA found one significant component in both ECM and VBM data (${ECM:\;p}_{corr}=2.9\times 10^{-2},\;AUC=0.71$; VBM: $p_{corr}=2.0\times 10^{-3},\;AUC=0.73$). PCA+CCA associated these two significant components at the 1st pair of canonical variates with canonical correlation ρ = 0.48. Parallel ICA found one significant component in VBM but not in the ECM data (ECM: *p*_*corr*_ = 0.10, *AUC* = 0.68; VBM: $p_{corr}=1.8\times 10^{-3},\;AUC=0.75$). The correlation between the most significant component in ECM and VBM was ρ = 0.27. sCCA found two significant ECM components and one VBM component (ECM: $p_{corr}=1.3\times 10^{-2},\;AUC=0.70$ and $p_{corr}=4.7\times 10^{-2},\;AUC=0.55$; VBM: $p_{corr}=1.6\times 10^{-2},\;AUC=0.70$). The correlation between the most significant component in ECM and VBM was ρ = 0.80. Among these four fusion methods, sPCA+CCA achieved the highest group classification accuracy 0.68, which was more than 99-percentile of the null distribution. The classification accuracy with concatenated ECM and VBM principal component scores *without fusion* as input features to QDA was 0.57.

**Table 1:** Measurements of the modulation profiles in sPCA+CCA, PCA+CCA, parallel ICA and sCCA.

**Methods**	**ECM**		**VBM**		**Correlation ρ**	**Classification accuracy**
	**p_corr_**	**AUC**	**p_corr_**	**AUC**		
sPCA+CCA	**3.4 × 10^−4^**	**0.78**	**2.6 × 10^−4^**	**0.81**	0.78	**0.68**
PCA+CCA	2.9 × 10^−2^	0.71	2.0 × 10^−3^	0.73	0.48	0.61
Parallel ICA	0.10	0.68	1.8 × 10^−3^	0.75	0.27	0.58
sCCA	1.3 × 10^−2^	0.70	1.6 × 10^−2^	0.70	**0.80**	0.60

*sCCA obtains the highest correlation due to overfitting, but has very low values in AUC. sPCA+CCA has largest AUC and a large correlation indicating that this method is superior to the other 3 methods. **p**_**corr**_ denotes the **p** value for group discrimination with multiple comparison correction. The group classification accuracy is obtained by running quadratic discriminant analysis on modulation profiles from both modalities. The most significant p-value and the largest value for each measurement are in bold font*.

**Figure 6:**
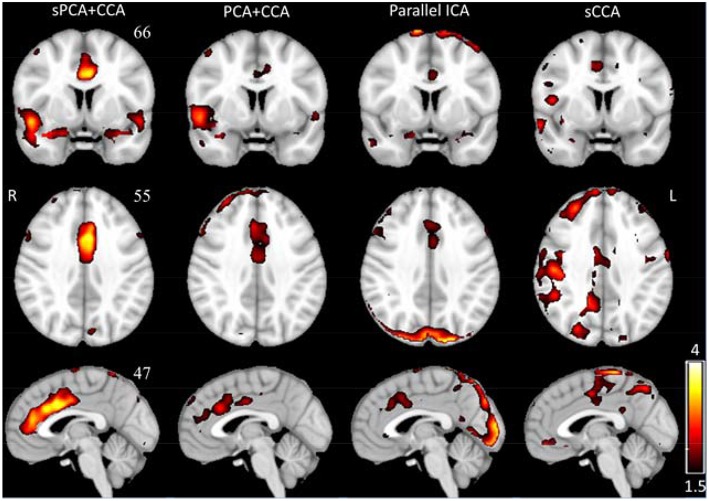
The most significant disease-related ECM z-score maps from sPCA+CCA, PCA+CCA, parallel ICA and sCCA. Maps are displayed in radiological convention (right is left and vice versa). All spatial maps are thresholded at z ≥ 1.5 except the map from parallel ICA that is thresholded at z ≥ 1. Parallel ICA would not show the anterior cingulate cortex if the map is thresholded at z ≥ 1.5.

The spatial patterns for these four fusion methods were also computed for sPCA+CCA and PCA+CCA by using Equation (11). ECM z-score spatial patterns corresponding to the most significant components in ***A***_*ECM*_ are shown in Figure 6. All spatial maps were thresholded at *z* ≥ 1.5 except the one from parallel ICA. The ECM spatial map from parallel ICA only showed an artifact on the brain boundary if thresholded at *z* ≥ 1.5, hence the threshold was lowered to *z* ≥ 1 for better interpretation. Anterior cingulate cortex (Bianciardi et al., 2009) was shown in the spatial patterns for all fusion methods. Both PCA+CCA and parallel ICA show some artifacts at the boundary of the brain. Bilateral superior temporal gyrus and bilateral amygdala were found in the ECM spatial pattern from sPCA+CCA.

**Figure 7:**
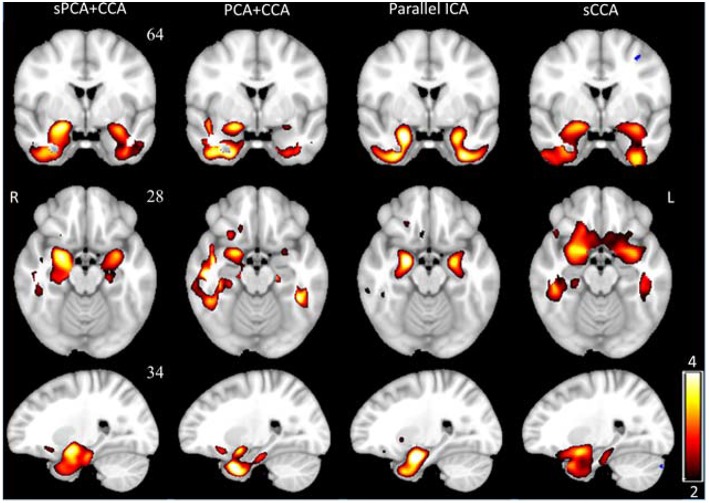
The most significant disease-related VBM z-score maps from sPCA+CCA, PCA+CCA, parallel ICA and sCCA. Maps are displayed in radiological convention (right is left and vice versa). All spatial maps were thresholded at z > 2. Note that PCA+CCA does not give a bilateral disease-related pattern.

VBM z-score maps corresponding to the most significant components in ***A***_*VBM*_ are shown in Figure 7. The spatial maps were thresholded at *z* ≥ 2. The VBM spatial maps are very similar except the one from PCA+CCA. In the VBM spatial maps, all fusion methods show gray matter atrophy in bilateral hippocampus and inferior temporal gyrus.

**Figure 8:**
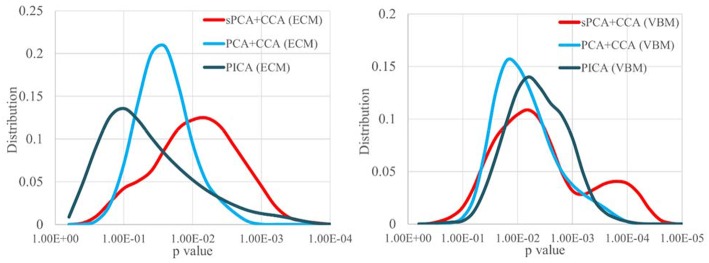
The distribution of *p*-values for group discrimination with the number of principal components ranging from 4 to 20. VBM dataset has more significant *p*-value than ECM dataset. The sPCA+CCA method has more significant group discrimination than PCA+CCA and parallel ICA.

Since the performance difference among these fusion methods shown in Figure 6 and Figure 7 may be affected by the number of remaining principal components, we have run sPCA+CCA, PCA+CCA and parallel ICA with the number of principal components ranging from 4 to 20 for both ECM and VBM datasets. For each fusion method, the most significant *p*-values for group discrimination with the number of principal components varying from 4 to 20 were recorded and the distribution of *p*-values is shown in Figure 8. The VBM datasets overall has more significant group difference than ECM datasets. Compared to PCA+CCA and parallel ICA, sPCA+CCA tends to have *p*-value more significant.

#### Correlation Between Disease-Related Modulation Profiles and β-Amyloid Measurement

**Figure 9:**
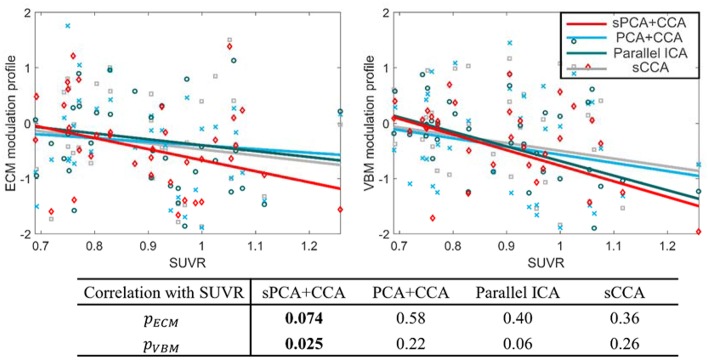
Correlation between the most significant modulation profiles in ECM and VBM modalities with SUVR. The significance *p*-values for ECM (*p*_*ECM*_) and VBM data (*p*_*VBM*_) in sPCA+CCA, PCA+CCA, parallel ICA and sCCA are also shown. The SUVR measures the content of ß-amyloid using PET scanning. The ECM modulation profile of the disease-related pattern measures the strength of functional connectivity. A more negative value indicates lower functional connectivity. The VBM modulation profile of the disease-related pattern relates to the amount of atrophy. More atrophy is present for negative values of the VBM modulation profile. Note that sPCA+CCA has the most significant negative correlation with SUVR.

The most disease-related modulation profile in ***A***_*ECM*_ and ***A***_*VBM*_ were correlated with SUVR, a measure of β-amyloid content calculated from the PET scans within 6 months of MRI scans. The correlation plots are shown in Figure 9. Each value in the ECM modulation profile measures the strength of functional connectivity for one subject, and a more negative value indicates lower functional connectivity. Similarly, each value in the VBM modulation profile measures the amount of atrophy for one subject, and a more negative value indicates more severe atrophy. Among these plots, only the VBM modulation profile in sPCA had a significant negative correlation with SUVR (*p* <0.05) and the other correlations were not significant. sPCA+CCA had the strongest correlation with SUVR in both ECM and VBM data.

## Discussion

To the best of our knowledge, our study is the first study proposing the sPCA+CCA method *and* comparing it with other methods for fusion analysis of multimodal brain imaging data. A novel split-sample cross-validation algorithm with *AIC* as selection criterion was validated for sPCA to determine the sparsity tuning parameter and the number of principal components. The sPCA+CCA fusion method extracts disease-related modulation profiles with the highest statistical power in real data. While sPCA and its variants were applied for noise elimination and functional segmentation in neuroimaging research (Ulfarsson and Solo, 2007; Ng et al., 2009; Khanna et al., 2015), to the best of our knowledge, this is the first study to implement and validate sPCA in fusion analysis.

### Properties of sPCA

Since sPCA is a sparse version of PCA, naturally they have some common properties. Both are linear techniques for dimensionality reduction. High-dimensional data is projected to a subspace spanned by the dominant principal component scores so that most of the variance in the original data is kept in a low-dimensional feature space. However, sPCA is different from PCA in terms of robustness, implementation, orthogonality, and computation.

1. *Robustness*: sPCA not only searches for the direction to maximize variance but also discriminates informative voxels from non-informative voxels as a data-driven approach. In other words, sPCA is useful when the number of features is large, while only a small proportion of them are informative. In many cases the salient features such as age- and disease-related features in the modalities are limited to only a few regions but not the entire brain. The sPCA method adjusts principal components by setting non-informative voxels to zero and hence obtains more robust scores (projection of original data on principal components) as the input to the following CCA analysis. In the sPCA+CCA fusion method, sPCA itself does not have discriminatory power. However, it was shown that sPCA is more robust against noise than conventional PCA. The similarity values *S*_*Y*_ and *S*_*V*_ in Figure 3 indicate that sPCA outperforms PCA in uncovering the true principal component scores and spatial maps, especially when the sparsity level is high. The robust scores from sPCA improve the subsequent CCA analysis to better link related modulation profiles and extract the corresponding spatial patterns in the data.
2. *Implementation*: Unlike PCA, which represents a standard eigenvalue problem, sPCA is a constrained optimization problem and optimized by an iterative algorithm. The objective function in sPCA is a biconvex function and is solved by optimizing two convex subproblems, both of which can be solved reliably and efficiently.
3. *Orthogonality*: The orthogonality no longer strictly holds when the *L*_1_ norm penalty term is added in sPCA. However, at the optimal sparsity tuning parameter, the mean absolute correlation with sign ignored between different principal components is 0.054, indicating that the principal components from sPCA are nearly orthogonal.
4. *Computation*: sPCA is more computationally intensive than standard PCA. Along with choosing the number of principal components as in PCA, sPCA also needs to specify the sparsity tuning parameter. Overestimated sparsity would be detrimental since informative voxels are also removed, and underestimated sparsity may not significantly improve the analysis. A grid search in PCA is carried out over the number of principal components, and a grid search in sPCA is carried out over a sparsity tuning parameter and the number of principal components. The grid search process exponentially increases computational time of sPCA because more parameters need to be optimized.

### Comparison of Fusion Analysis

Fusion analysis was carried out with simulated and real data. In the simulations, the sPCA+CCA method has improved performance over PCA+CCA by about 10% at sparsity level 70%. We have tested these two fusion methods at different sparsity levels and found that the improvement decreases with lower sparsity level until the performance difference becomes negligible when the sparsity level is about 30%. We would like to point out that parallel ICA does not lead to orthogonal components because orthogonality is not strictly enforced unlike CCA-based fusion methods. Thus, the simulation generated with an orthogonality condition is biased toward CCA-based fusion methods and explains why parallel ICA does not perform well in our simulation. In contrast, generating data without the assumption of orthogonality would make the simulation more biased toward parallel ICA. Among the four fusion methods considered, sCCA overestimates the correlation between modalities and also has low similarity. Unlike sCCA having original voxel-wise input features, sPCA+CCA along with PCA+CCA and parallel ICA reduces the data dimension *before* fusing modalities and thus possibly may discard some correlated features that have low variance. The voxel-wise input features to sCCA, however, are much larger than the number of samples. For example, the number of non-zero features in sCCA is at the order of a thousand, while the number of input features to CCA in sPCA+CCA is of the order of ten. The elastic-net penalty as a sparsity constraint may not be sufficient to alleviate an overestimated canonical correlation relationship and thus sPCA+CCA still outperforms sCCA in both simulated and real data.

In real data, the proposed sPCA+CCA method has the most significant disease-related modulation profiles in both modalities and the highest group classification accuracy. Compared to the accuracy obtained with principal component scores as input, using the modulation profiles as input have improved classification accuracy for all considered fusion methods. ACC is found to be disease-related by all fusion methods in ECM data. A decreased functional connectivity of ACC was consistent with the findings in previous resting-state fMRI studies (Rombouts et al., 2005; Sheline et al., 2009) and ACC was also found to be affected in MCI subjects by other imaging techniques, such as single photon emission computed tomography (SPECT) and structural MRI studies (Huang et al., 2002; Karas et al., 2004). sPCA+CCA found that the amygdala and the superior temporal gyrus *bilaterally*, in addition to ACC, are important disease-related regions in the ECM data. Decreased functional connectivity of the amygdala and superior temporal gyrus in MCI or Alzheimer's disease subjects were also found in previous fMRI studies (Celone and Calhoun, 2006; Liu and Zhang, 2012; Yao et al., 2013), and are consistent with our results. In the VBM disease-related spatial maps, hippocampus and inferior temporal gyrus are found to have more atrophy in all fusion methods. The hippocampus is a critical region in the limbic system that is involved in motivation, emotion, learning and memory. Atrophy in the hippocampus is closely related to early symptoms in AD patients, such as short-term memory loss and disorientation. Early hippocampal atrophy is an established biomarker of AD (Jack et al., 1999). We also found that the inferior temporal gyrus is affected in MCI. This region is essential in face, pattern, and object recognition, and may already be affected in early-stage MCI subjects (Whitwell et al., 2008).

The disease-related modulation profiles from sPCA+CCA, PCA+CCA, parallel ICA and sCCA were correlated with the measure of β-amyloid, i.e., SUVR (Figure 9). Only sPCA+CCA found significant correlation with SUVR in VBM data but not in ECM data. The ECM modulation profile from sPCA+CCA, however, had strongest correlation with SUVR among all of ECM modulation profiles. A more negative value in the ECM modulation profile indicates lower functional connectivity, and a more negative value in the VBM modulation profile indicates more severe atrophy in the disease-related patterns. Since SUVR is used for longitudinal analyses in MCI (Landau et al., 2014), the disease-related spatial pattern and corresponding modulation profile from our fusion method potentially can be used to monitor disease severity.

Similar to other fusion methods, sPCA+CCA has its own assumptions and limitations. From the simulation and the formulation of sPCA+CCA, we illustrate that the CCA step enforces orthogonality on the modulation profile for each modality. In addition, implementing sparsity assumes that the associated effect between modalities is distributed locally instead of globally across the brain. This assumption is realistic because in amnestic MCI or early AD not the entire brain shows atrophy or loss of functional connectivity, but the disease state is limited to sparse brain regions such as the inferior temporal lobes and the posterior cingulate cortex. Enforcing sparsity in fusion analysis is applicable to many neurological diseases in their early stages. On a computational level, enforcing sparsity significantly increases computational time. Computations were run on a Dell workstation with 2 Intel Xeon E5-2643 processors. This is different from parallel ICA and PCA+CCA, where the computation takes only minutes to carry out a fusion analysis. In contrast, sPCA+CCA needs approximately 12 h to complete the analysis, and sCCA needs ~21 h.

### Extension of sPCA+CCA

We would like to emphasize that sPCA also can be applied to other CCA-based fusion methods such as multiset CCA (Correa et al., 2010) and CCA+jICA (Sui et al., 2010, 2011). Unlike CCA that associates only two modalities, multiset CCA is applied when more than two modalities are considered for fusion analysis. In the CCA+jICA method, joint ICA is carried out after CCA to maximize the independence among joint components and to prevent CCA from failing to separate sources. Since PCA is also required in these two methods for dimensionality reduction, sPCA can be incorporated into these methods as well. If the structural information in the brain map is pre-specified, more sophisticated sparse constraints such as structural lasso (Simon et al., 2013; Lin et al., 2014) can potentially be used in sparse fusion methods including sPCA+CCA and sCCA. However, more advanced methods are beyond the scope of the current study.

### Limitations and Future Study

The proposed sPCA method has two limitations. First, as in PCA, sPCA preserves the global structure of the data but ignores the Euclidean structure of image space and hence may lead to discrete non-zero voxels in sparse principal components. Second, the property of orthogonality between principal components does not strictly hold because of the lasso penalty used in sPCA. Furthermore, the issue of missing data is not addressed in this study. Some subjects may only have one imaging modality available or have data with partial brain coverage, while some fusion methods have been developed to address this issue (Xiang et al., 2014; Pan et al., 2018), current sPCA+CCA framework cannot use subjects with missing data.

Other dimension reduction methods, such as the locality preserving projection method (He and Niyogi, 2003), were studied extensively in pattern recognition. However, the performance of more sophisticated dimension reduction techniques for neuroimaging studies is unknown. The auto encoder related methods (Bengio, 2009) are currently of high interest in the deep learning research community. This method is appealing for handling non-linear systems and could replace the linear PCA algorithm. One critical reason for requiring dimension reduction in CCA-based fusion analysis is that the number of features in standard CCA algorithm cannot be more than the number of observations. If CCA itself can be revised to select features adaptively and avoid the singularity problem arising from too many features, then the dimension reduction preprocessing step may not be required.

## Conclusion

We have proposed a sPCA algorithm for data fusion and compared sPCA with three different state-of-the-art fusion methods. We evaluated how well these fusion methods associate related patterns in different modalities and correlated the result from fusion analysis with ß-amyloid measurement (SUVR). We found that sPCA can significantly reduce the impact of non-informative voxels and improve statistical power for uncovering disease-related patterns. The sPCA+CCA method not only achieves the best group discrimination but also has the strongest correlation with the SUVR measurement. In summary, sPCA is a powerful method for sparse regularization and dimensionality reduction, completely data-driven, and self-adaptive without experts' intervention.

## Author Contributions

ZY and DC: conception and design of the study. ZY, XZ, KS, VM, SB, and DC: analysis and interpretation of data. CB: data management and quality control. DC and ZY: drafting the article. ZY, XZ, CB, KS, VM, SB, and DC: revising it critically for important intellectual content and final approval of the version to be submitted.

### Conflict of Interest Statement

The authors declare that the research was conducted in the absence of any commercial or financial relationships that could be construed as a potential conflict of interest.
