# Supplementary material for: Performing Sparse Regularization and Dimension Reduction Simultaneously in Multimodal Data Fusion
Source: Front Neurosci. 2019 Jul 3;13:642. doi: 10.3389/fnins.2019.00642 (PMC6618346; doi:10.3389/fnins.2019.00642)
Supplement: Supplementary file 1 [file Data_Sheet_1.docx]

**APPENDIX A**

Let $d_{k}$ denote the elements of the diagonal matrix $\boldsymbol{D}$**,** and $\boldsymbol{u}_{k}$,$\boldsymbol{v}_{k}$ the column $k$ of the matrices $\boldsymbol{U,}$ $\boldsymbol{V}$, respectively. The function to be minimized is given by $f_{obj}\left( \boldsymbol{U},\boldsymbol{D},\boldsymbol{V} \right)=\left\| \boldsymbol{X}-\boldsymbol{UD}\boldsymbol{V}^{T} \right\|_{F}^{2}$, where the square of the Frobenius norm of a matrix $\boldsymbol{A=(}a_{ij}\boldsymbol{)}$ is defined by $\left\| \boldsymbol{A} \right\|_{F}^{2}=\sum_{i} \sum_{j} {{|a}_{ij}\boldsymbol{|}}^{\boldsymbol{2}}=tr(\boldsymbol{A}^{\boldsymbol{T}}\boldsymbol{A)}$. We can simplify the expression for $f_{obj}$ and have the following expression

${f_{obj}=\left\| \boldsymbol{X}-\boldsymbol{UD}\boldsymbol{V}^{T} \right\|}_{F}^{2}=tr\left( \left( \boldsymbol{X}-\boldsymbol{UD}\boldsymbol{V}^{T} \right)^{T}\left( \boldsymbol{X}-\boldsymbol{UD}\boldsymbol{V}^{T} \right) \right)$ (A1)

$$=-2tr\left( \boldsymbol{X}^{T}\boldsymbol{UD}\boldsymbol{V}^{T} \right)+tr\left( \boldsymbol{VD}\boldsymbol{U}^{T}\boldsymbol{UD}\boldsymbol{V}^{T} \right)+\left\| \boldsymbol{X} \right\|_{F}^{2}$$

$$=\sum_{k=1}^{K} d_{k}^{2}-2\mathrm{tr}\left( \boldsymbol{D}\boldsymbol{U}^{T}\boldsymbol{XV} \right)+\left\| \boldsymbol{X} \right\|_{F}^{2}$$

$$=\sum_{k=1}^{K} d_{k}^{2}-2\sum_{k=1}^{K} d_{k}\boldsymbol{u}_{k}^{T}\boldsymbol{X}\boldsymbol{v}_{k}+\left\| \boldsymbol{X} \right\|_{F}^{2}.$$

Since $\left\| \boldsymbol{X} \right\|_{F}^{2}$ is a constant, $f_{obj}$ is a convex function if only $d_{k}$ is considered as independent variable and the optimal $d_{k}$ satisfies $d_{k}=\boldsymbol{u}_{k}^{T}\boldsymbol{X}\boldsymbol{v}_{k}$. Then, $f_{obj}\left( d_{1},\ldots,d_{K} \right)=-\sum_{k=1}^{K} d_{k}^{2}+\left\| \boldsymbol{X} \right\|_{F}^{2}$, and the minimization of $f\left( d_{1},\ldots,d_{K} \right)$ is equivalent of maximizing $\sum_{k=1}^{K} d_{k}^{2}$ with $d_{k}=\boldsymbol{u}_{k}^{T}\boldsymbol{X}\boldsymbol{v}_{k}$. For a rank 1 approximation i.e.$K=1$, minimization of $f_{obj}\left( d_{1} \right)$ is equivalent to maximize $d_{1}=\boldsymbol{u}_{1}^{T}\boldsymbol{X}\boldsymbol{v}_{1}$.

**APPENDIX B**

The objective function in PCA is

$f_{\boldsymbol{obj}}\left( d\boldsymbol{,u,v} \right)\boldsymbol{=}\frac{1}{2}\left\| \boldsymbol{X-du}\boldsymbol{v}^{\boldsymbol{T}} \right\|_{F}^{2}\boldsymbol{,} s.t. \left\| \boldsymbol{u} \right\|_{2}^{2}\boldsymbol{=}1\boldsymbol{,}\left\| \boldsymbol{v} \right\|_{2}^{2}\boldsymbol{=}1\boldsymbol{.}$ (B1)

During the recursively iterative algorithm, e.g., fix $\boldsymbol{u}$ and optimize $\boldsymbol{v}$, the Lagrangian can be written as

$L\left( \boldsymbol{v;}\lambda_{2} \right)\boldsymbol{=}\frac{1}{2}\left\| \boldsymbol{X-}d\boldsymbol{u}\boldsymbol{v}^{\boldsymbol{T}} \right\|_{F}^{2}\boldsymbol{+}\lambda_{2}\left\| \boldsymbol{v} \right\|_{2}^{2}, \lambda_{2}\geq0$, (B2)

where the second term on the right side is the $L_{2}$ penalty. When sparsity is introduced to PCA, the $L_{1}$ penalty is added and the Lagrangian becomes

$L\left( \boldsymbol{v;}\lambda_{1}, \lambda_{2} \right)\boldsymbol{=}\frac{1}{2}\left\| \boldsymbol{X-du}\boldsymbol{v}^{\boldsymbol{T}} \right\|_{F}^{2}\boldsymbol{+}\lambda_{2}\left\| \boldsymbol{v} \right\|_{2}^{2}\boldsymbol{+}\lambda_{1}\left\| \boldsymbol{v} \right\|_{1}\boldsymbol{,} \lambda_{2}\geq0, \lambda_{1}\geq0$, (B3)

with the *elastic* penalty (a convex combination of $L_{1}$ and $L_{2}$ penalties). For this reason, sPCA has a unique solution even when the number of features is much larger than the number of subjects (Zou et al., 2006). We would like to emphasize that minimizing the Lagrangian with $L_{2}$ norm of $\boldsymbol{v}$ as in Eq. (B3) gives the same principal components as the iteration algorithm described in section 2.1 that forces $\boldsymbol{v}$ to be of unit norm. A previous study (Zou et al., 2006) showed that the solution of Eq. (B2) or Eq. (B3) at any positive $\lambda_{2}$ is the principal component with only a scaling difference. Hence, forcing $\boldsymbol{v}$ to be unit norm in sPCA is the same as varying the $\lambda_{2}$ parameter in the elastic penalty of Eq.(B3) to achieve the unit norm without impacting the final solution.

**APPENDIX C**

The optimization problem is given by

$\min_{v} -\boldsymbol{a}^{\boldsymbol{T}}\boldsymbol{v} s.t. \left\| \boldsymbol{v} \right\|_{2}^{2}=1, \left\| \boldsymbol{v} \right\|_{1}\leq c.$ (C1)

The Lagrangian of the above problem is

$L\left( \boldsymbol{v};\lambda,\mu\right)=-\boldsymbol{a}^{T}\boldsymbol{v}+\lambda\left\| \boldsymbol{v} \right\|_{2}^{2}+\mu\left\| \boldsymbol{v} \right\|_{1},$ (C2)

where $\lambda>0$,$\mu\geq0$ are Lagrange parameters. The Karush-Kuhn-Tucker equations associated with the above optimization problem are given by

$-\boldsymbol{a}+2\lambda\boldsymbol{v}+\mu\boldsymbol{\partial}\left\| \boldsymbol{v} \right\|_{1}=0,$ (C3.1)

$\lambda\left( \left\| \boldsymbol{v} \right\|_{2}^{2}-1 \right)=0,$ (C3.2)

$\mu\left( \left\| \boldsymbol{v} \right\|_{1}-c \right)=0.$ (C3.3)

The vector operator $\boldsymbol{\partial}$ acting on the non-differentiable function $\left\| \boldsymbol{v} \right\|_{1}$ in Eq.(C3.1) represents the subgradient vector. In our case, the subgradient for the $i$-th component is:

$\partial_{i}\left\| \boldsymbol{v} \right\|_{1}=\left\{ \begin{matrix} \text{sign}\left( v_{i} \right) \mathrm{if}v_{i}\neq0 \\ \text{interval }\left[ -1,1 \right] \mathrm{if}v_{i}=0 \end{matrix} \right.$. (C4)

Solving Eq. (C3.1) for the $i$-th component of $\boldsymbol{v}$ when $v_{i}\neq0$ results in $v_{i}=\frac{a_{i}-\mu\mathrm{sign}\left( v_{i} \right)}{2\lambda}$. For $a_{i}>\mu$, it follows that $v_{i}>0,$ and $\mathrm{sign}\left( v_{i} \right)=\mathrm{sign}\left( a_{i} \right)$. Similarly, if $a_{i}<-\mu$, it follows that $v_{i}<0$, and $\mathrm{sign}\left( v_{i} \right)=\mathrm{sign}\left( a_{i} \right)$. If $v_{i}=0$, it follows from Eq. (B3.1) that $-a_{i}+\mu\left[ -1,1 \right]=-a_{i}+\left[ -\mu,\mu\right]=0$ only has a solution when $a_{i}\in\left[ -\mu,\mu\right]$. Putting these scenarios together, we can write

$v_{i}=\left\{ \begin{matrix} \frac{a_{i}-\mu\mathrm{sign}\left( a_{i} \right)}{2\lambda} & \mathrm{if}\left| a_{i} \right|>\mu\\ 0 & \mathrm{if}\left| a_{i} \right|\leq\mu\end{matrix} \right..$ (C5)

Equation (C3.2) is satisfied by normalizing the vector $\boldsymbol{v}$**.** Thus, our final result is

$v_{i}=\left\{ \begin{matrix} \frac{a_{i}-\mu\mathrm{sign}\left( a_{i} \right)}{\left\| \boldsymbol{v} \right\|_{2}} & \mathrm{if}\left| a_{i} \right|>\mu\\ 0 & \mathrm{if}\left| a_{i} \right|\leq\mu\end{matrix} \right. .$ (C6)

Using the definition for the soft threshold function $S\left( a_{i},\mu\right)=\text{sign}\left( a_{i} \right)\max\left( 0, \left| a_{i} \right|-\mu\right)$ (see Fig.C1 below for an example), $a_{i}-\mu\mathrm{sign}\left( a_{i} \right)=\text{sign}\left( a_{i} \right)\max\left( 0, \left| a_{i} \right|-\mu\right)=S\left( a_{i},\mu\right)$ when $\left| a_{i} \right|>\mu$. Then, we can write the solution for vector $\boldsymbol{v}$ as

$\boldsymbol{v}=\frac{S(\boldsymbol{a},\mu)}{\left\| S\left( \boldsymbol{a},\mu\right) \right\|_{2}} .$ (C7)

where $S(\boldsymbol{a},\mu)$ acts on each component of vector $\boldsymbol{a}$**.** The condition $\mu\left( \left\| \boldsymbol{v} \right\|_{1}-c \right)=0$ requires $\left\| \boldsymbol{v} \right\|_{1}=c$; otherwise $\mu=0$. The solution to Eq. (C7) is obtained by testing if $\mu=0$ leads to a solution with $\left\| \boldsymbol{v} \right\|_{1}\leq c$. If this is not the case, then a $\mu>0$ is found by a binary search algorithm such that $\left\| \boldsymbol{v} \right\|_{1}=c$. A brief pseudocode for $K$ factors sPCA on an arbitrary matrix $\boldsymbol{X}$ is given below:

For $k=1, \ldots, K$;

- 1. Initialize $\boldsymbol{u}$ and normalize such that $\boldsymbol{u}=\frac{\boldsymbol{u}}{\left\| \boldsymbol{u} \right\|_{2}}$;
  2. Iterate until converge: $\boldsymbol{v}=\frac{S(\boldsymbol{X}^{T}\boldsymbol{u}, \mu)}{\left\| S\left( \boldsymbol{X}^{T}\boldsymbol{u}, \mu\right) \right\|_{2}}$; try $\mu=0$, if the result satisfies $\left\| \boldsymbol{v} \right\|_{1}\leq c$ stop; otherwise, $\mu$ is chosen to be a positive constant that $\left\| \boldsymbol{v} \right\|_{1}=c$. Update $\boldsymbol{u}$ as $\boldsymbol{u}=\frac{\boldsymbol{Xv}}{\left\| \boldsymbol{Xv} \right\|_{2}}$;
  3. $d_{k}=\boldsymbol{u}_{k}^{T}\boldsymbol{X}\boldsymbol{v}_{k}$;
  4. Update $\boldsymbol{X}$ as $\boldsymbol{X}\leftarrow\boldsymbol{X}-d_{k}\boldsymbol{u}_{k}\boldsymbol{v}_{k}^{T}$;

End for loop


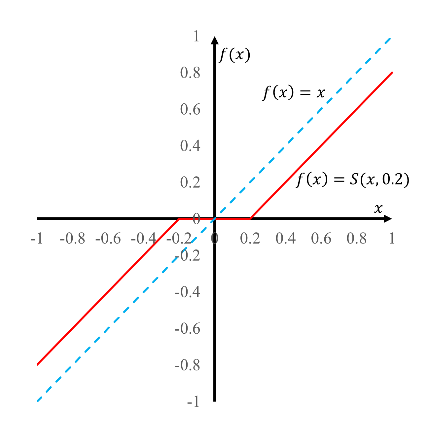


**Figure C1. An example of a soft-threshold function** $\boldsymbol{S(x,}\mathbf{0.2}\boldsymbol{)}$**.**

**APPENDIX D**

The Akaike Information Criterion is given by $AIC=-2\mathcal{L+}2K$, where $\mathcal{L}$ is the maximum log-likelihood of a Gaussian distribution and $K$ is the number of free parameters associated with the complexity of the model. The log-likelihood at its maximum can be written as

$\mathcal{L}\left( \boldsymbol{R},\boldsymbol{\Sigma} \right)=-\frac{1}{2}\log\left( {det}^{*}(2\pi\boldsymbol{\Sigma)} \right)-\frac{1}{2}\sum_{i=1}^{\eta} \boldsymbol{R}_{i}\boldsymbol{\Sigma}^{+}\boldsymbol{R}_{i}^{T}$, (D1)

where $\boldsymbol{R}$ is the residual between original data and the reconstructed data, $\eta$ is the number of samples, and $\boldsymbol{\Sigma}$ is the covariance matrix of features (Hazewinkel, 2001). Since the covariance matrix is highly degenerate, the pseudo-inverse $\boldsymbol{\Sigma}^{+}$ and pseudo-determinant ${det}^{*}$ are used in Eq.D1. Fig.D1a and Fig.D1b shows the AIC calculation diagrams for one fold in the split-sample and revised Witten’s $F$-fold cross-validation methods, respectively. In the split-sample method, sPCA is carried out with $\boldsymbol{X}^{\boldsymbol{(}\bar{f}\boldsymbol{)}}$, then the obtained principal components $\boldsymbol{V}^{\left( \bar{f}) \right.}$ are used to fit $\boldsymbol{X}^{(f)}$ by a linear regression model, and finally the AIC for the *f*-fold dataset, denoted as $AIC^{\left( f \right)}$, can be calculated with the residual $\boldsymbol{R}$. In the revised Witten’s imputation method, the 1/*F* randomly removed elements in $\boldsymbol{X}^{(\bar{f})}$ are first filled with the mean value in each column as ${\tilde{\boldsymbol{X}}}^{(\bar{f})}$. Then sPCA is applied on ${\tilde{\boldsymbol{X}}}^{(\bar{f})}$ and a matrix having the same dimension as the original data $\boldsymbol{X}$ can be constructed as $\hat{\boldsymbol{X}}=\boldsymbol{UD}\boldsymbol{V}^{T}$. Finally the residual $\boldsymbol{R}$ is calculated by subtracting $\boldsymbol{X}$ from $\hat{\boldsymbol{X}}$ with only the elements in $\boldsymbol{X}^{(f)}$ considered and all other elements assigned to be zero. For each fold dataset, the grid search algorithm is applied over sparsity tuning parameter *c* and the number of principal components *K*. Let {$c^{(f)}, K^{(f)}$} denote the parameters having minimum AIC for the *f* fold dataset, the optimal sparsity parameter $c^{*}$ is defined as the average over $c^{(f)}, f=1,\ldots, F$, and the optimal number of principal components $K^{*}$ is the rounded integer of the average over $K^{(f)}, f=1,\ldots,F$.

To evaluate which of the two different cross-validation methods have better performance, we have generated a set of simulated data with 5 principal components and sparsity levels 30%, 50% and 70%. 10-fold cross-validation was carried out with PSNR of 2 dB, 4 dB and 6 dB. The simulation was repeated 40 times. Both cross-validation methods can accurately estimate the number of principal components $K^{*}$, however, they estimate the sparsity level differently. The histograms over estimated sparsity level for the split-sample method (red histogram) and the revised Witten’s imputation method (blue histogram) were shown in Fig.D2. The black triangle “” indicates the simulated sparsity level and the green triangle “” indicates the mean sparsity level. When the PSNR was low, the revised Witten’s imputation method sometimes failed to estimate the correct sparsity level, especially for the case with sparsity level=0.3, 0.5 and PSNR=2dB. The Split-sample method underestimated the sparsity level at sparsity level 0.3 but had a more unbiased result when the sparsity level was at higher values 0.5 and 0.7. The revised Witten’s imputation method, however, had more scattered results and severely overestimated sparsity when PSNR was 2 dB and also tended to overestimate sparsity in other cases, such as for sparsity level=0.5 and PSNR=6dB. The split-sample method overall outperformed the revised Witten’s imputation method, and hence the split-sample method was used in this study for parameter selection.


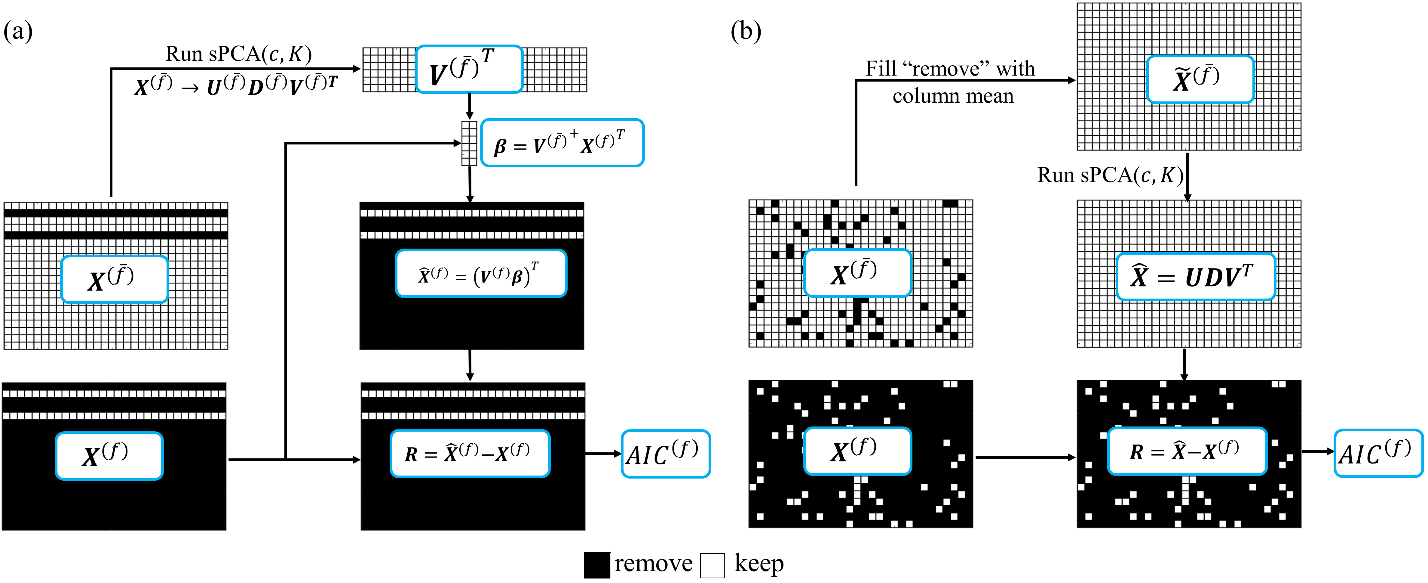


**Figure D1. Two different cross-validation methods used in sPCA to select parameters. (a) Split-sample method. (b) Revised Witten’s imputation method.**


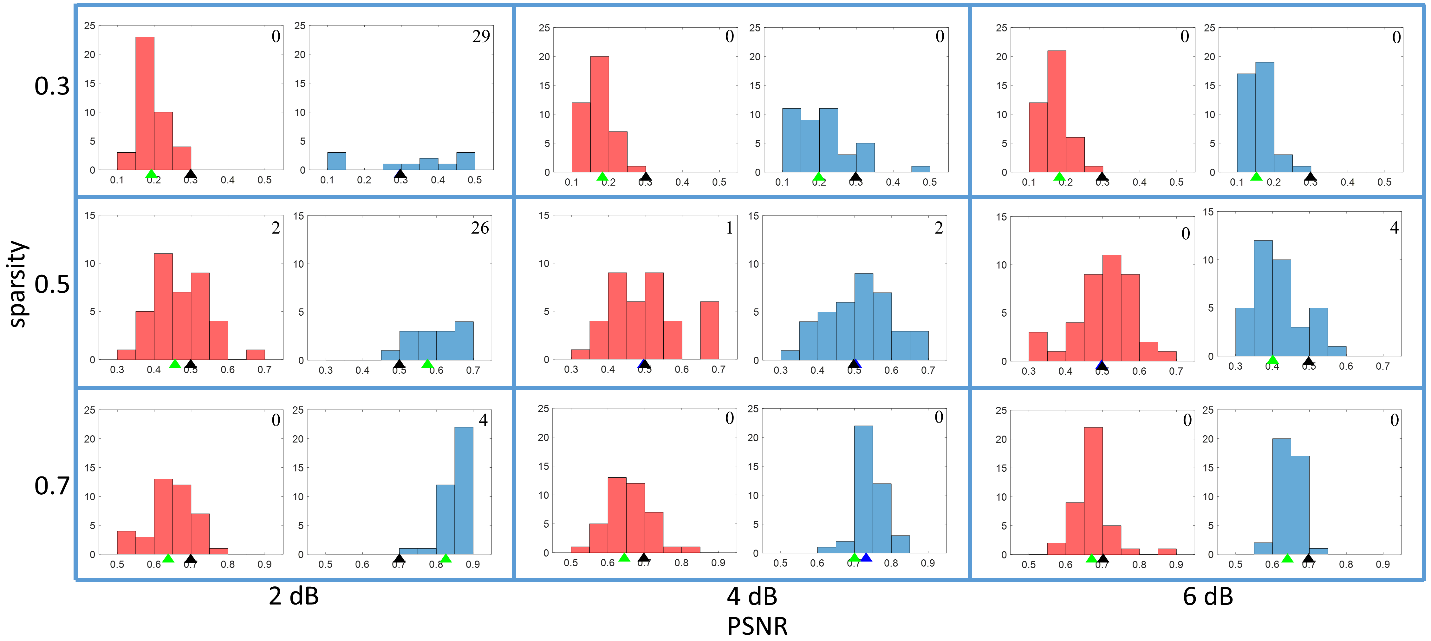


**Figure D2: Histograms of estimated sparsity levels from split-sample method (red histogram) and revised Witten’s method (blue histogram). The simulation was carried out 40 times with true sparsity levels 0.3, 0.5, and 0.7 under PSNR 2 dB, 4 dB and 6 dB. The black triangle “” indicates the simulated sparsity level and the green triangle “” indicates the mean sparsity level. The number at the right top corner of each histogram denotes how many times the cross-validation failed to estimate the correct sparsity level. The split-sample method underestimates the sparsity level when the sparsity level is low but shows a more unbiased estimation when the sparsity level is higher. Compared to the revised Witten’s method, the split-sample method is more stable in estimating the sparsity level. Overall, the split-sample method outperforms the revised Witten’s method.**
